# Functionalized Bisphenol A-Based Polymer for High-Performance Structural Supercapacitor Composites

**DOI:** 10.3390/polym17172380

**Published:** 2025-08-31

**Authors:** Jayani Anurangi, Janitha Jeewantha, Hazem Shebl, Madhubhashitha Herath, Jayantha Epaarachchi

**Affiliations:** 1 School of Engineering, Faculty of Health Engineering and Sciences, University of Southern Queensland, Toowoomba, QLD 4350, Australia; jayani.anurangi@unisq.edu.au (J.A.); janitha.jeewantha@unisq.edu.au (J.J.); hazem.shebl@unisq.edu.au (H.S.); 2 Centre for Future Materials, Institute for Advanced Engineering and Space Sciences, University of Southern Queensland, Toowoomba, QLD 4350, Australia; m.herath@hull.ac.uk; 3 Department of Biosystems Technology, Faculty of Technological Studies, Uva Wellassa University of Sri Lanka, Passara Road, Badulla 90000, Sri Lanka; 4 School of Engineering, University of Hull, Hull HU6 7RX, UK

**Keywords:** Bisphenol A polymer, structural supercapacitor, elevated temperatures, graphene-nanoplatelet-coated carbon fibre electrode, finite element modelling

## Abstract

Over the last few decades, polymer composites have been rapidly making inroads in critical applications of electrical storage devices such as batteries and supercapacitors. Structural supercapacitor composites (SSCs) have emerged as multifunctional materials capable of storing energy while bearing mechanical loads, offering lightweight and compact solutions for energy systems. This study investigates the functionalization of Bisphenol A-based thermosetting polymers with ionic liquids, aiming to synthesize dual-functional structural electrolytes for SSC fabrication. A multifunctional sandwich structure was subsequently fabricated, in which the fabricated SSC served as the core layer, bonded between two structurally robust outer skins. The core layer was fabricated using carbon fibre layers coated with 10% graphene nanoplatelets (GNPs), while the skin layers contained 0.25% GNPs dispersed in the resin matrix. The developed device demonstrated stable operation up to 85 °C, achieving a specific capacitance of 57.28 mFcm^−2^ and an energy density of 179 mWhm^−2^ at room temperature. The performance doubled at 85 °C, maintaining excellent capacitance retentions across all experimented temperatures. The flexural strength of the developed sandwich SSC at elevated temperature (at 85 °C) was 71 MPa, which exceeds the minimum requirement for roofing sheets as specified in Australian building standard AS 4040.1 (Methods of testing sheet roof and wall cladding, Method 1: Resistance to concentrated loads). Finite element analysis (FEA) was performed using Abaqus CAE to evaluate structural integrity under mechanical loading and predict damage initiation zones under service conditions. The simulation was based on Hashin’s failure criteria and demonstrated reasonable accuracy. This research highlights the potential of multifunctional polymer composite systems in renewable energy infrastructure, offering a robust and energy-efficient material solution aligned with circular economy and sustainability goals.

## 1. Introduction

The rapid increase in global population and industrial development has led to an unprecedented energy demand that cannot be met by the existing fossil fuel reserves [1,2]. The continuous expansion of fossil fuel combustion significantly contributes to environmental pollution, substantially impacting climate change and global sustainability [3,4]. As a result, there is a growing need to transition toward clean and sustainable energy sources such as solar, wind, and hydro power [5,6]. However, the primary challenge associated with those renewable energy resources is their intermittent and fluctuating nature compared to conventional energy resources [7,8]. Therefore, developing high-performance energy storage devices is crucial to ensure a continuous and reliable energy supply before adopting renewable energy resources.

Recent advancements in polymer science have enabled the development of multifunctional polymers that combine energy storage functionality with structural performance, offering promising solutions for lightweight and compact energy systems [9,10,11,12]. In this regards, Bisphenol A-based thermosetting polymers have gained considerable attention as ideal candidates for structural composite applications [9,13,14,15]. These polymers can serve as active components within energy storage systems, when functionalized with electrochemically active materials. A promising strategy to integrate the electrochemical properties of Bisphenol A-based polymers is the development of structural electrolytes by the incorporation of ionic liquids into the epoxy matrix [16,17]. This integration results in forming a structural electrolyte with a bi-continuous structure, wherein the ionic liquid is confined within the crosslinked resin network [18,19]. This bi-continuous structure enables the Bisphenol A-based polymer to store energy while simultaneously retaining its mechanical load-bearing capability.

In recent years, the integration of structural polymers with electrochemical functionalities has made a breakthrough for the development of structural supercapacitor composites (SSCs). SSCs offer a unique opportunity to embed energy storage functions directly into structural components, significantly reducing the weight and volume penalties associated with traditional standalone energy storage devices. Carbon-fibre-reinforced composites (CFRCs) are particularly attractive for this purpose, as they can be engineered to deliver both structural reinforcement and electrochemical activity [16,20]. In such systems, carbon fibre (CF) serves as the electrode material, while the polymer matrix functionalized with electrochemically active materials acts as the electrolyte. However, pristine CF inherently exhibits limited electrochemical performance due to its chemical inertness and low active surface area [21,22]. Consequently, significant research has focused on improving CF surface characteristics to enhance SSC performance [23]. In this context, various carbon nanomaterials, such as activated carbon [24], carbon nanotubes (CNT) [25,26,27], carbon aerogel [28], graphene nanoflakes [22], graphene aerogel (GA) [29], and graphene nanoplatelets (GNPs) [13,30] have been investigated for their electrochemical properties, and some of these materials can be coated onto CF fabrics to enhance the SSC performance.

Recent findings have identified GNP as one of the most suitable materials for coating on CF surfaces for high electrical, mechanical, and thermal properties [13,30,31]. For example, Javaid et al. improved the specific capacitance value from 8.9 mFcm^−3^ to 118.7 mFcm^−3^ and normalized the in-plane shear modulus value from 1.7 GPa to 3.1 GPa after interleaving GNP between CFs and solid polymer electrolyte [13]. In addition, another study demonstrated that GNP improved the capacitance from 3.1 mFg^−1^ to 9.6 mFg^−1^ and Young modulus from 23.7 GPa to 24 GPa [32]. However, these studies have demonstrated that focusing solely on developing individual components presents a significant challenge to advancing this technology, as it often results in a trade-off between mechanical and electrochemical performance.

Considering the facts mentioned above, SSCs based on structural electrolytes have limitations in their properties due to the conflicting requirements of ion transport and mechanical rigidity. Therefore, recent research has focused on exploring novel fabrication techniques and designs to address this drawback rather than developing new materials for structural supercapacitor components.

In this regard, Sun et al. fabricated a hybrid laminated structural composite to improve the mechanical and electrical properties simultaneously [33]. The outer layer consists of four layers of sandwiched kevlar fabric/epoxy prepregs, while the inner layer consists of a thin interleaf of carbon fibre/solid electrolyte supercapacitor. The developed model demonstrated high electrochemical and mechanical performance, achieving a specific capacitance of 0.872 mFg^−1^, an energy density of 0.08 mWhkg^−1^, and a power density of 9.2 mWkg^−1^ at a current density of 0.05 Acm^−3^. The hybrid laminate flexural strength and flexural modulus were measured as 192 MPa and 9.3 GPa, respectively. However, delamination cracks between the supercapacitor interleaf and kevlar/epoxy lamina were initiated when the external load increased to a certain level due to the material inhomogeneity-induced stress concentration.

Mapleback et al. introduced a new design methodology for SSCs to eliminate the poor ionic conductivity due to solid electrolytes [34]. They proposed localising the supercapacitor electrolyte within the composite structure, enabling high electrochemical performance in localized areas while maintaining high mechanical performance elsewhere. However, the results highlight the need for significant improvements, as the devices have not yet met the performance level required for practical applications. Thus, further research is essential to enhance the performance and expand the application potential of structural supercapacitor devices.

On the other hand, a robust structural supercapacitor must deliver high electromechanical performance and withstand extreme operating conditions since many engineering applications are exposed to various environmental conditions [18]. As stated by Yadav et al. [35], the structural power composite should resist electrical and mechanical cycling, and temperature extremes, and exhibit fire resistance and impact and damage tolerance.

Temperature is an important parameter determining supercapacitor performance and cycle life [36,37]. While elevated temperatures can promote electrochemical reactions, they degrade the performance of supercapacitors over time [18,38,39]. However, comprehensive studies on the electrochemical performance of SSCs at elevated temperatures are lacking [40]. In addition, the residual strength of fibre-reinforced composites drastically reduces at high temperatures [41,42]. However, there is a lack of studies evaluating the performance of SSCs at various operating conditions and developed SSCs for applications in different surroundings.

This study proposes a novel structural supercapacitor based on a sandwich composite structure designed to enhance electrochemical performance and mechanical strength, addressing the challenges of developing high-performance structural supercapacitor composites. The developed composite comprises a high-performance electrochemical core layer bonded to two thin, durable skin layers. A parallelly connected GNP-coated double-cell configuration was utilized to fabricate the core layer. The sandwich SSC was tested under cyclic voltammetry (CV), galvanostatic charge–discharge (GCD), and three-point bending load at three different temperature levels (25, 65, and 85 °C) to assess its feasibility for practical applications at in-service temperatures of up to 85 °C. Moreover, finite element analysis (FEA) using Abaqus CAE was conducted to assess the structural integrity of the sandwich SSC and predict damage initiation under mechanical loading. The model incorporated Hashin’s failure criteria, widely recognized for predicting failure mechanisms in fibre-reinforced composites, enabling the identification of damage initiation and propagation zones within the structure. The simulation results showed good agreement with experimental observations, validating the reliability and predictive capability of the FEA approach in evaluating the structural performance of the sandwich SSC.

## 2. Materials and Methods

### 2.1. Materials

Carbon fibre fabric (200 gsm, twill weave, purchased from ATL Composites, Gold Coast, Australia) and E-glass fibre fabric (120 gsm, twill weave, purchased from ALT Composites, Australia) were used as the reinforcements in this study, and GNP (G-750, Sigma Aldrich, Sydney, Australia) acted as the nanomaterial. The PTFE (60 wt% dispersion in H_2_O, Sigma Aldrich, Australia) and Triton X-100 (Sigma Aldrich, Australia) were utilized as the binder and the surfactant, respectively, when making GNP slurry with IPA solvent (purity: 98%, FG, Sigma Aldrich, Australia).

Bisphenol A polymer (Diglycidylether of bisphenol (DGEBA- Araldite GY 191, Huntsman Ltd., The Woodlands, TX, USA)) was used as the epoxy resin, and triethylenetetramine (TETA, purity: 97%, purchased from Sigma Aldrich, Australia) was used as the hardener. 1-Ethyl-3-methylimidazolium bis (trifluoro methylsulfonyl) imide (EMITFSI, purity: 98%, purchased from Sigma Aldrich, Australia) was the ionic liquid. Bis (trifluoromethane) sulfonamide lithium salt (LiTFSI, purchased from Sigma Aldrich, Australia), propylene carbonate (PC, purity: 99.7%, purchased from Sigma Aldrich, Australia), and industrial-grade TiO_2_ (purity: 99%, 20–40 nm, XFNANO China, Nanjing, China) were mixed with the ionic liquid of EMITFSI for preparing liquid electrolyte (LE). Techniglue structural epoxy with a fast hardener (purchased from ATL Composites, Australia) was selected for bonding composite layers. All chemical reagents were used without further purification.

### 2.2. Functionalization of Polymer

Bisphenol A-based polymer (DGEBA) was functionalized with ionic conductivity by incorporating an ionic liquid (EMIMTFSI). Firstly, to prepare the liquid electrolyte (LE), 11.8 g of EMIMTFSI, 0.1 g of propylene carbonate, and 1 g of LiTFSI were magnetically stirred for 30 min. Subsequently, 3.65 g of DGEBA was added to the LE solution and mixed for an additional 20 min. Then, 0.5 g of TiO_2_ was incorporated and the mixture was stirred for a further 30 min. Finally, TETA hardener was added at a stoichiometric ratio, and the mixture was stirred for 5 min to obtain the structural electrolyte (SE) solution. The resulting SE solution comprised 75% liquid electrolyte and 25% structural resin matrix. The morphology of the functionalized DGEBA-based structural electrolyte was examined using scanning electron microscopy (SEM). In addition, its thermal and mechanical properties were characterized in our previous study to evaluate its suitability for multifunctional applications under elevated-temperature conditions.

### 2.3. Structure of Sandwich SSC

**Figure 1:**
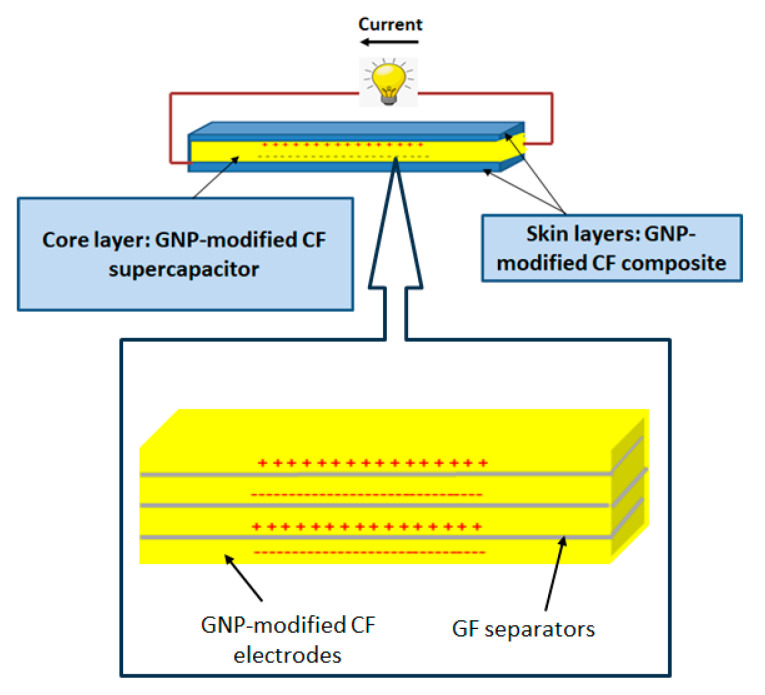
Structure of sandwich structural supercapacitor composite.

The sandwich SSC was fabricated as shown in Figure 1. A GNP-modified carbon fibre supercapacitor developed by a previous study was used as the high-performance electrochemical core layer. In that study, GNP-coated CF fabrics were used as electrodes, and the coating procedure and characterization of the GNP-coated electrodes were detailed [31]. The surface morphology and uniformity of the GNP coating were studied by scanning electron microscopy (SEM), and surface wettability was evaluated using contact angle analysis.

A GNP-enhanced composite, wherein GNPs were incorporated into the matrix, was fabricated to serve as the skin layers. The electrical performance of the sandwich SSC depends on the electrochemical properties of the core layer; the properties of all constituent materials influence the mechanical performance. Thus, the sandwich structure provides the flexibility to tailor specific properties, and therefore, a better balance of performance is expected from this structure.

### 2.4. Fabrication of Structural Supercapacitor Core Layer

**Figure 2:**
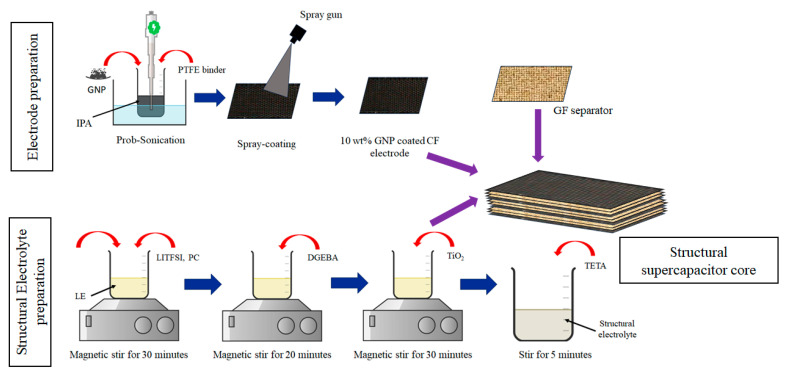
Schematic of structural supercapacitor core fabrication process.

GNP slurry was prepared as shown in Figure 2 and coated on the CF fabric using a spray gun. The active GNP loading (GNP+PTFE binder) on the CF fabric was estimated to be approximately 10 wt%. It was calculated from the mass increment of the CF-GNP fabric concerning the pristine CF fabric based on Equation (S1). Then, parallelly connected GNP-coated double-cell SSC was fabricated with the configuration of CF-GNP/GF/GF/CF-GNP/GF/GF/CF-GNP/GF/GF/CF-GNP. Finally, the fabricated double-cell structural supercapacitor core was cured at room temperature (RT 25 °C) for 24 h and post-cured at 85 °C for 3 h. This was the supercapacitor functional core layer of the sandwich composite; its thickness was approximately 2.5 mm. The Supplementary Materials detail the preparation of the GNP slurry, spray coating, and fabrication of the structural supercapacitor core.

### 2.5. Fabrication of Skin Layer

**Figure 3:**
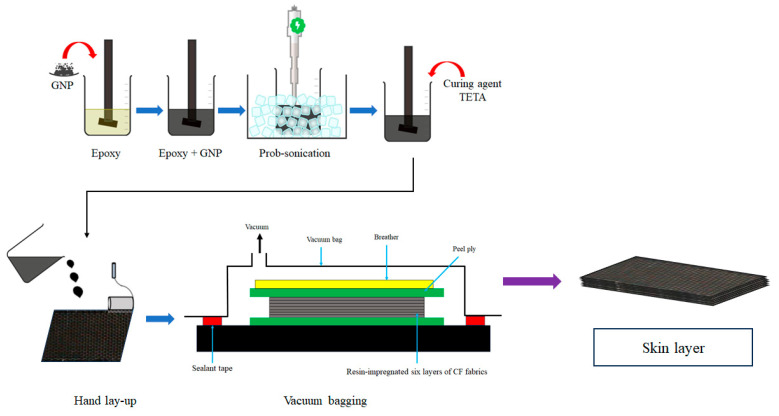
Schematic of skin layer fabrication process.

The GNP-modified resin matrix was prepared (Figure 3) by incorporating 0.25 wt% of GNPs Araldite GY 9708-3, followed by adding a TETA hardener. Next, GNP-modified CF laminates were fabricated using six layers of CF fabric through the hand lay-up technique, followed by a vacuum bagging process, as shown in Figure S1. The laminates were cured at room temperature for 24 h, post-cured at 85 °C for 8 h in an oven, and used as skin layers of the sandwich composite.

### 2.6. Fabrication of Supercapacitor Functional Sandwich Composite

**Figure 4:**
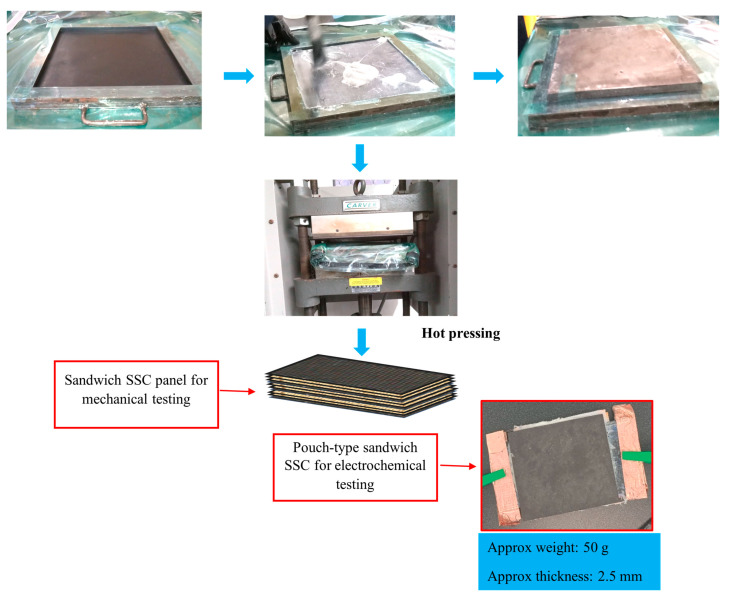
Schematic of sandwich SSC fabrication process.

As shown in Figure 4, a sandwich structure with a supercapacitor functional core and two GNP-modified CF skins was fabricated using the hot-pressing method. The layers were bonded using Techniglue structural epoxy with a fast hardener. A 300 mm × 300 mm × 6.5 mm panel was fabricated for flexural testing, and specimens were cut using a water jet cutter. For electrochemical testing, a symmetric pouch-type supercapacitor functional sandwich composite with an effective surface area of 50 mm × 50 mm [Figure S2] was fabricated, as detailed in the Supplementary Materials.

### 2.7. Electrochemical Testing

All electrochemical measurements were taken using an Autolab electrochemical interface instrument (PGSTAT 302N with Nova 2.1.4). CV tests were performed at scan rates of 5 mVs^−1^ and 100 mVs^−1^ for comparison of electrochemical behaviour, while GCD tests at a 0.24 mAcm^−2^ current density evaluated the specific areal capacitance. Areal capacitance was evaluated in this study to enable consistent comparison with similar structural supercapacitors and to allow direct estimation of total capacitance when scaling the device for practical applications. All experiments were conducted in two electrode configurations at 25 °C, 65 °C, and 85 °C temperatures, as shown in Figure S3. The samples were conditioned in an oven at the respective temperatures for 2 h before conducting tests at elevated temperatures (65 °C and 85 °C). The measurements were recorded while maintaining the corresponding temperature, as shown in Figure S3b. Additionally, the fabricated SSCs were subjected to 1000 cycles of CV at 5 mVs^−1^ to study the effect of operating temperature on its lifespan. Finally, the functionality of the developed sandwich SSC was demonstrated by powering a commercial LED bulb.

### 2.8. Flexural Testing

#### 2.8.1. Experimental Analysis

**Figure 5:**
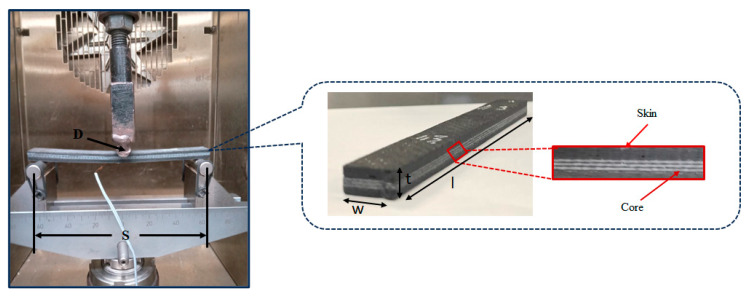
Experimental set-up for three-point bending test.

As shown in Figure 5, three-point bending tests were performed on specimens (135 mm (l) × 15 mm (w) × 6.5 mm (t)) following the ASTM D790 standard at 25 °C, 65 °C, and 85 °C temperatures. The load was applied at a crosshead speed of 1 mmmin^−1^ at the centre using a uni-axial testing machine equipped with a 10 kN load cell with a loading nose diameter (D) of 5 mm. The number of specimens tested for each condition was determined according to ASTM D790, and the average values of these replicates were reported. Strain gauges (gauge length: 5 mm, gauge factor: 2.16, FLA-5-23-1L, Tokyo Sokki Kenkyujo Co., Ltd., Tokyo, Japan) were attached at the mid-span of the bottom skin face to record the strain data. The load, deflection, and strains for each test were recorded.

#### 2.8.2. Finite Element Analysis

**Figure 6:**
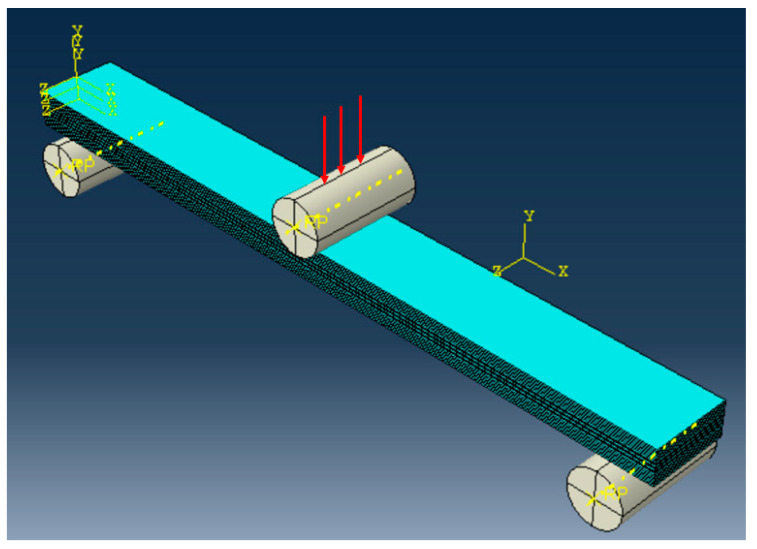
FEA model with six CF plies in each skin layer and a core layer consisting of four CF plies and six GF plies.

FEA analysis for flexural behaviour of the sandwich SSC was conducted using Abaqus CAE for a specimen (135 mm (l) × 15 mm (w) × 6.5 mm (t)), as shown in Figure 5. The material properties of core and skin layers of the sandwich SSC, evaluated in previous studies (as listed in Table 1), were used as input data for the FEM model implementation. The applied boundary conditions are shown in Figure 6, and a lateral load was applied at the centre of the laminate along the *y*-axis. A detailed description of the FEA model elements is provided in Table 2, with additional information on the analysis available in the Supplementary Materials. The analysis continued until the first significant failure occurred in the sandwich composite.

**Figure 7:**
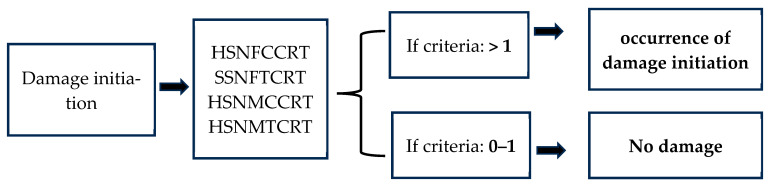
Schematic illustration of Hashin’s criteria.

Damage initiation was analysed based on Hashin’s failure criteria, a widely accepted method for analysing the damage behaviour of fibre-reinforced composites [43,44,45,46]. This model is particularly suitable for predicting failure modes under various stress conditions in hybrid composites, considering the interaction of different fibres with the matrix [47]. The criteria can evaluate the damage initiation based on the achievement of initiation parameters. Moreover, the analysis can be extended to assess the damage progression. Hashin’s criteria consider four failure types: fibre tension (HSNFTCRT), fibre compression (HSNFCCRT), matrix tension (HSNMTCRT), and matrix compression (HSNMCCRT); the following relationships (Equations (1)–(4)) account for each type. Each damage initiation parameter ranges from 0 to 1, and when any parameter reaches 1, it indicates the occurrence of damage initiation, as described in Figure 7.

Fibre in tension:

(1) $$F_{f}^{t}=\;{\left.\left(\;\frac{\sigma_{11}}{X^{T}}\right.\right)}^{2\;}+\;{\left.\alpha \left(\;\frac{\tau_{12}}{S^{L}}\right.\right)}^{2\;}$$

Fibre in compression:

(2) $$F_{f}^{c}=\;{\left.\left(\;\frac{\sigma_{11}}{X^{C}}\right.\right)}^{2\;}$$

Matrix in tension:

(3) $$F_{m}^{t}=\;{\left.\left(\;\frac{\sigma_{22}}{Y^{T}}\right.\right)}^{2\;}+\;{\left.\left(\;\frac{\tau_{12}}{S^{L}}\right.\right)}^{2\;}$$

The matrix in compression:

(4) $$F_{m}^{C}=\;{\left.\left(\;\frac{\sigma_{22}}{Y^{L}}\right.\right)}^{2\;}+\left[{\left.\left(\;\frac{Y^{C}}{{2S}^{T}}\right.\right)}^{2\;}-1\right]\frac{\sigma_{22}}{Y^{C}}+\;{\left.\left(\;\frac{\tau_{12}}{S^{L}}\right.\right)}^{2\;}$$

In equations, $F_{f}^{t}$, $F_{f}^{c}$, $F_{m,}^{t}$, and $F_{m}^{C}$ represent the damage initiation parameter of each mode, respectively. *X^T^* and *X^C^* are tension and compression strength in the longitudinal direction, while *Y^T^* and *Y^C^* are tension and compression strength in transverse directions. *S^L^* and *S^T^* denote the shear strengths in longitudinal and transverse directions. α is a factor that represents shear contribution in the tensile fibre direction (0 or 1).

## 3. Results and Discussion

### 3.1. Characterization of Bisphenol A-Based Structural Electrolyte

**Figure 8:**
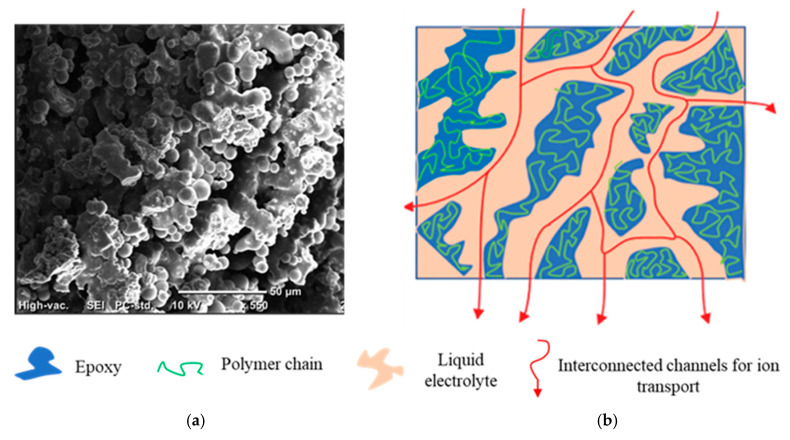
(**a**) Bi-continuous structure in DGEBA after incorporation of liquid electrolyte, (**b**) schematic representation of interconnected channel formation in DGEBA facilitating ionic conductivity [16].

The addition of ionic liquid into the polymer system forms a bi-continuous structure within the structural electrolyte, leading to significant modifications in the morphology of the final material, as shown in Figure 8a. During the curing process, the liquid electrolyte forms a distinct phase within the structural resin matrix, which is subsequently encapsulated and confined by the crosslinked epoxy network. This encapsulated phase imbues additional functionalities to the epoxy system. Specifically, the liquid electrolyte phase facilitates ionic conductivity through interconnected channels formed within the matrix as shown in Figure 8b.

The thermal properties of the functionalized DGEBA-based structural electrolyte were previously evaluated using thermogravimetric analysis (TGA), confirming its thermal stability under elevated temperature conditions [18]. The electrolyte displayed a two-stage decomposition profile, with an onset temperature of approximately 330 °C. Notably, the mass loss between room temperature (25 °C) and the operational upper limit (85 °C) was less than 1%, indicating excellent thermal stability within the functional temperature range of the device.

Dynamic mechanical analysis (DMA) from the same study [18] revealed a peak tan δ at 82 °C and an onset of viscoelastic transition at approximately 51 °C. These results demonstrate that the structural electrolyte retains its mechanical stiffness and structural integrity throughout the operating temperature range (25–85 °C), confirming its suitability for multifunctional applications in elevated-temperature environments.

### 3.2. Electrochemical Measurements

**Figure 9:**
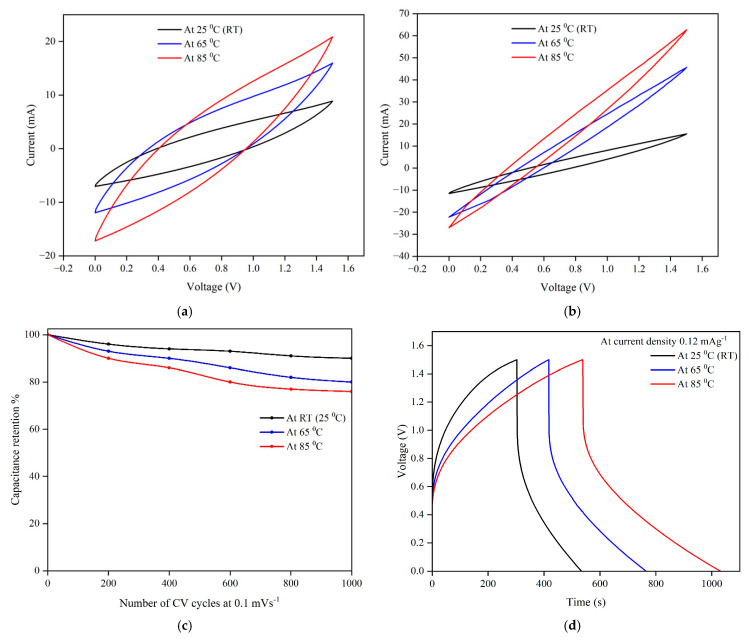
Electrochemical performance: (**a**) CV test results at 5 mVs^−1^, (**b**) CV test results at 10 mVs^−1^, (**c**) cyclic stability, and (**d**) GCD test results at 0.24 mAcm^−2^.

CV curves (Figure 9) were analysed to compare the electrochemical behaviour at three different temperatures. The area under the CV curve gradually increases with increased temperature due to the higher ionic conductivity resulting from faster reaction kinetics and higher ionic mobility. As shown in Figure 9a,b, the quasi-rectangular shape of all CV curves indicates that the developed device can operate within the tested temperature range without undergoing redox reactions. The specific capacitance values (C_sp, CV_) are calculated from the area inside the curve using the formula in Equation (S2) and are presented in Table S1. An increase in capacitance values was noted with rising temperature. The elevated temperature may lead to prolonged cycling and reduced lifetime of the sandwich SSC. However, long cycle life is an important parameter in practical applications. Thus, the SSC’s cyclic stability was analysed by running 1000 cycles of CV at a scan rate of 5 mVs^−1^ at three temperatures. Figure 9c illustrates the specific capacitance retention vs. cycle numbers. Only a minor variation was observed in specific capacitance after 1000 CV cycles, with 90%, 80%, and 76% capacitance retentions at room temperature (25 °C), 65 °C, and 85 °C, respectively. This proves the SSC can maintain remarkably high cyclic stability in the testing temperature range. The long cycle life of the supercapacitor can be attributed to the high electrochemical stability of the GNP at elevated temperatures.

The electrochemical behaviour of the device was further evaluated through GCD measurements, as illustrated in Figure 9d. The capacitive performance of the devices at different temperatures was calculated according to the GCD profile. The specific capacitance (C_sp, GCD_) and energy density (E_sp, GCD_) were calculated using Equations (S3) and (S4), respectively. It demonstrates a gradual increase with increasing temperature. At a current density of 0.24 mAcm^−2^, it exhibited a specific capacitance of 57.28 mFcm^−2^ and an energy density of 179 mWhm^−2^ at room temperature (25 °C). With increasing temperature, capacitance and energy density increased to 113 mFcm^−2^ and 267.4 mWhm^−2^ at 65 °C and further to 113.56 mFcm^−2^ and 354.9 mWhm^−2^ at 85 °C, demonstrating a twofold increase compared to room temperature. The increase in energy density with temperature can be attributed to variations in ionic conductivity and electrochemical behaviour at elevated temperatures, while the relationship between temperature and energy density is slightly nonlinear.

**Figure 10:**
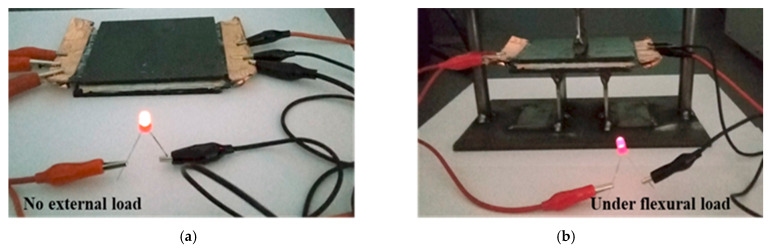
Lighting up a LED (**a**) without loading, (**b**) with three-point loading.

Finally, the fabricated sandwich SSC was used to power a commercial LED (Standard red, 5 mm, 2.3 V, 20 mA nominal current, 30 mA maximum current) to demonstrate the stability of device operation. First, the SSC was charged using a laboratory power supply of up to 5 V for 15 min and we measured the duration that it powered the LED. The successful LED operation for 10 min without external loading [Figure 10a] and with flexural loading [Figure 10b] confirmed the functionality of the developed structural supercapacitor. These results indicate that the developed supercapacitor has a promising potential for energy storage while supporting mechanical loads.

### 3.3. Flexural Behaviour of the Composite

#### 3.3.1. Performance at Room Temperature

##### Experimental Results

**Figure 11:**
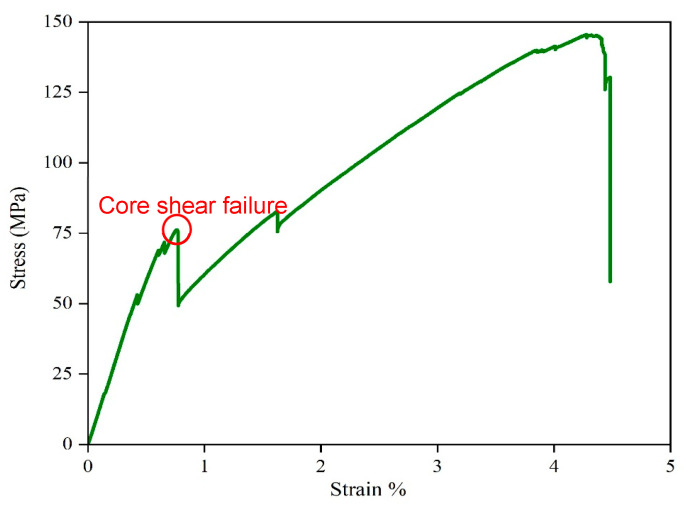
Stress–strain curve at room temperature.

**Figure 12:**
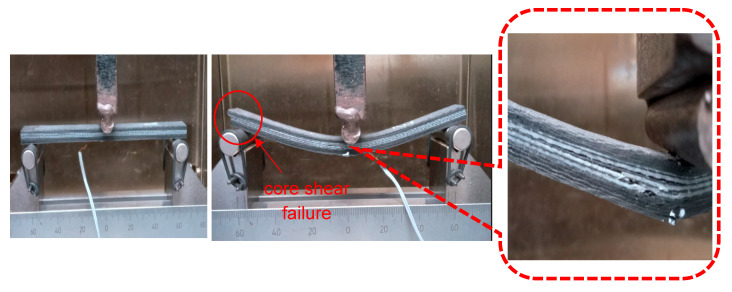
Failure progress of sandwich SSC.

The flexural data were analysed to evaluate the mechanical strength of the sandwich SSC, and Figure 11 illustrates the stress–strain curves at room temperature. As depicted in Figure 12, a progressive failure mechanism was observed in the composite under flexural loading. The first significant failure happened at a strain of 0.8%, attributed to core shear failure in the core layer. Surface cracks on the top and bottom surfaces were observed at the first failure point. Subsequently, multiple progressive failures at various strain levels happened due to the failure of different layers in the core when increasing the load. This behaviour is attributed to the mismatch in mechanical properties between the carbon fibre electrodes and glass fibre separators in the core layer [48]. In addition, interlaminar shear stresses and non-uniform stress distribution at the interfaces within the core led to deamination, further contributing to the complex failure behaviour beyond the first failure point. Following the multiple progressive failures, the device completely failed its load-carrying capacity at 4.5% strain at a maximum strength of 146 MPa. To provide a comparison for this study, a summary of the performance of various structural supercapacitors from the literature is presented in Table 3 and compared with the results of this work.

##### Finite Element Analysis Results

The flexural test coupon was modelled using Abaqus CAE finite element software and validated through a comprehensive experimental programme. The model was validated until the first significant failure, and the sandwich composite’s damage initiation was studied based on Hashin’s failure criteria based on the values presented in Table S2.

##### Loading and Constraints/Boundary Condition and Contact Setting

The FEA model of the sandwich SSC under the three-point bending includes a loading nose and two support noses modelled using a discrete rigid body. Perfect bonding was assumed between the skin and core layers using surface-based tie contact, as no debonding failure was observed in the experiment. The model contains solid FE mesh with 12,240 elements in each skin and 20,250 elements in the core. Finally, a convergence test was performed by varying the mesh size, and the results were found to be within 5%, confirming the numerical stability of the simulation.

##### Data Validation and Prediction

**Figure 13:**
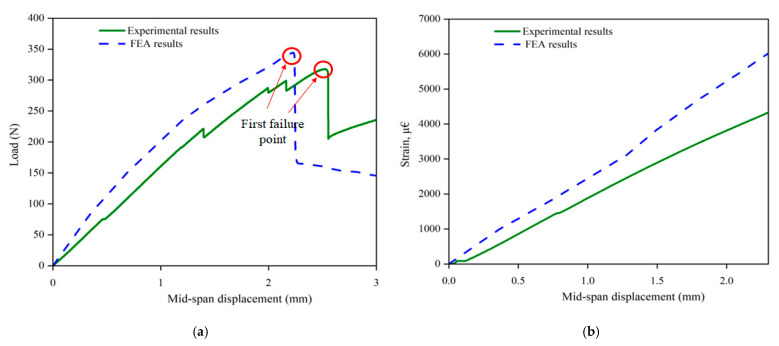
Experimental/numerical comparison: (**a**) load–displacement plot, (**b**) comparison of FEA strain data with experimental data.

The developed model was validated with the load–displacement data and the strain data from the bottom surface of the specimen, as shown in Figure 13. The results indicate a reasonable accuracy in the developed model for the elastic region up to the first failure point. However, the proposed model shows limited compatibility beyond the elastic region, highlighting the need for detailed failure analysis to accurately characterize the flexural behaviour beyond the failure point. Therefore, progressive failure analysis with damage evolution law until the material completely fails is recommended for future advancement of this work for detailed analysis of the material behaviour.

##### Hashin’s Failure Modes and Locations

**Figure 14:**
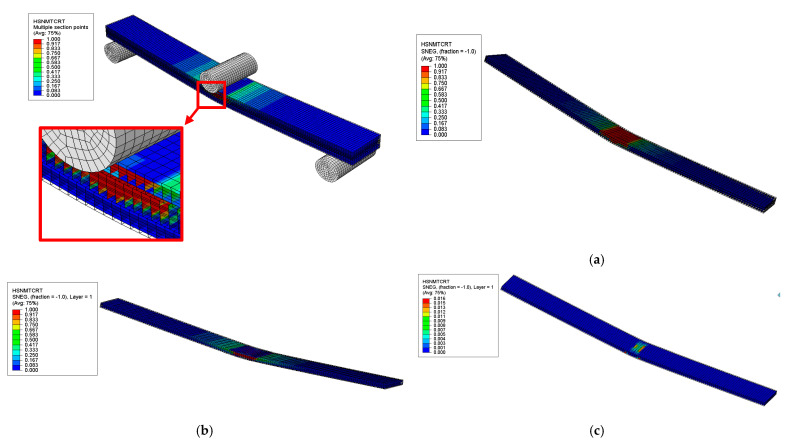
Results of HSNMTCRT failure mode of sandwich SSC: (**a**) core layer—initiation of failure observed, (**b**) top skin layer—initiation of failure observed, (**c**) bottom skin layer—no failure.

**Figure 15:**
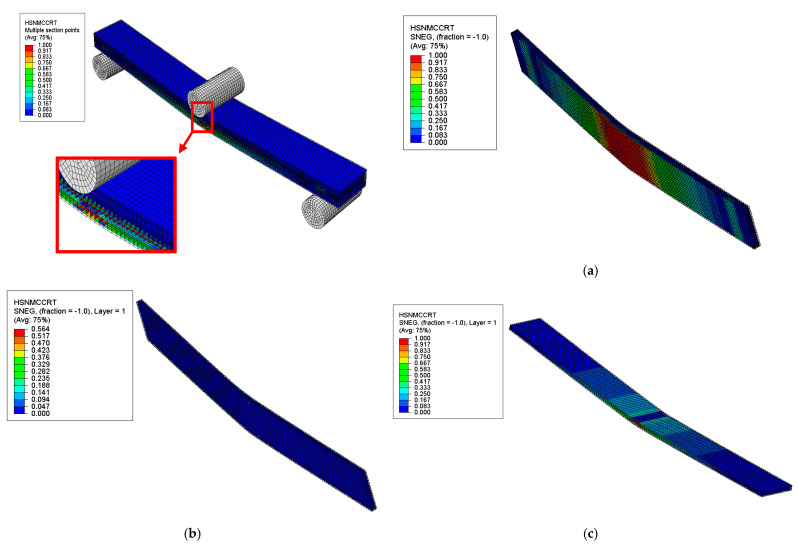
Results of HNSMCCRT failure mode of sandwich SSC: (**a**) core layer—initiation of failure observed, (**b**) top skin layer—no failure, (**c**) bottom skin layer—initiation of failure observed.

**Figure 16:**
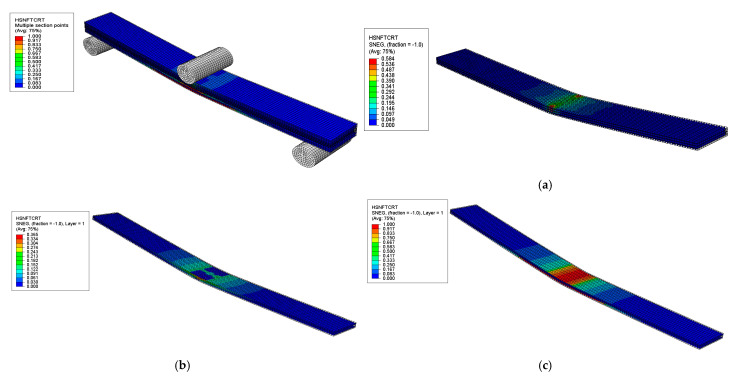
Results of HSNFTCRT failure mode of sandwich SSC: (**a**) core layer—no failure, (**b**) top skin layer—no failure, (**c**) bottom skin layer—initiation of failure observed.

**Figure 17:**
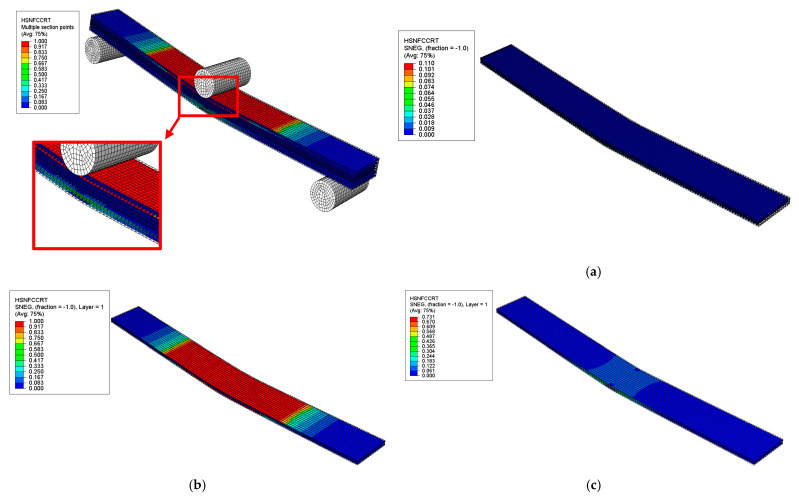
Results of HSNFCCRT failure mode of sandwich SSC: (**a**) core layer—no failure, (**b**) top skin layer—initiation of failure observed, (**c**) bottom skin layer—no failure.

The failure modes and locations of failures in the sandwich composite were investigated based on Hashin’s failure criteria at the onset of the first significant failure, as shown in Figure 14, Figure 15, Figure 16 and Figure 17. The results showed that different plies failed in various failure modes in the skin and core layers. The first significant failure occurred at 2.5 strain, as shown in the load–deflection curve.

#### 3.3.2. Performance at Elevated Temperatures

**Figure 18:**
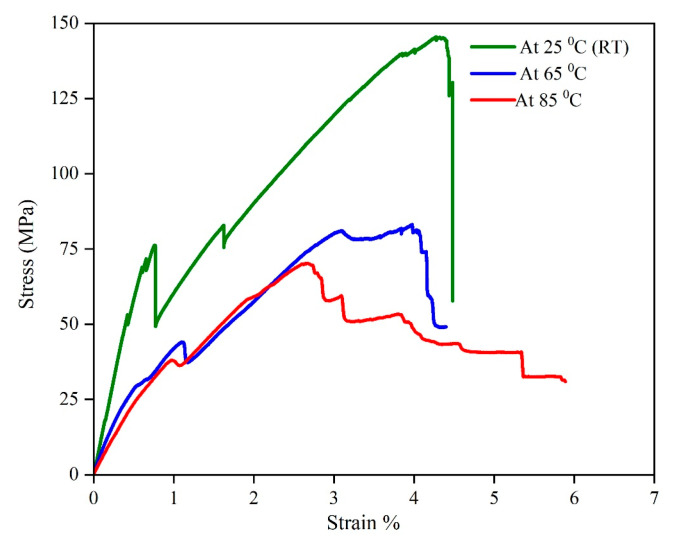
Stress–strain curves at different temperatures.

The flexural behaviour of the developed device was studied at elevated temperatures and illustrated in Figure 18. The ultimate strength values gradually decrease with increasing temperatures, reducing by 43% and 51% when temperatures rise to 65 °C and 85 °C. The mechanical performance losses at 85 °C are mainly due to the resin softening after passing the glass transition region of the structural electrolyte used for fabricating the core [18]. However, the developed material exhibited 71 MPA strength when the temperature reached 65 °C.

### 3.4. Proof of Concept: Mechanical Performance and Practical Implementation

The flexural strength of the developed composite panel is a critical parameter for real-world applications such as roofing sheets, as these structures must withstand bending loads caused by various external impacts. Therefore, to ensure its feasibility for real-world applications, the composite panel was validated against the requirements specified in Australian Standard AS 4040.1 (Methods of testing sheet roof and wall cladding, Method 1: Resistance to concentrated loads).

According to the calculations based on this standard, the minimum required flexural strength for a flat roofing sheet is 14 MPa. The experimental results demonstrated that the developed panel significantly exceeds this threshold, even when exposed to an in-service temperature of 85 °C, confirming its mechanical reliability under operational conditions.

Furthermore, the flexural strength of the developed panel is well within the range of commercially available roofing sheets, which typically exhibit a strength of approximately 50 MPa. Those findings indicated that the developed composite panel meets the requirements for practical applications, confirming its potential for use in civil engineering applications.

## 4. Conclusions

This study successfully demonstrated the development of a multifunctional sandwich SSC incorporating a functionalized Bisphenol A-based thermosetting polymer. It further addresses a critical gap by developing and validating a device capable of maintaining both electrochemical and mechanical stability at elevated temperatures. The device demonstrated stable operation up to 85 °C, with a specific capacitance of 57.28 mFcm^−2^ and an energy density of 179 mWhm^−2^ at room temperature, doubling its performance at elevated temperatures while maintaining excellent capacitance retention. Flexural strength remained robust, decreasing from 146 MPa at 25 °C to 71 MPa at 85 °C, surpassing the minimum strength requirements for structural applications. Thus, the performance of the developed SSC was comparable to that of commercial alternatives, confirming its practical viability. Moreover, the developed FEA accurately predicted damage initiation, supporting its mechanical reliability. In summary, this research advances the development of multifunctional energy storage materials by (i) presenting a novel multifunctional sandwich SSC with improved energy storage capability, capable of reliable operation at elevated temperature up to 85 C, which has not been previously reported; (ii) demonstrating structural performance aligned with civil engineering applications; and (iii) integrating experimental and numerical approaches for comprehensive evaluation. These findings not only expand the application potential of SSCs in renewable energy infrastructure but also open a new pathway for designing sustainable, lightweight, and durable structural energy storage systems in line with circular economy principles.

## Figures and Tables

**Table 1:** Static mechanical properties used in the FEA model, obtained by the experimental characterization of constituents.

Constituents		Elastic Moduli (GPa)	Shear Moduli (GPa)	Poisson’s Ratio
Skin		E1: 39	G1: 25	Nu12: 0.20
		E2: 20	G2: 25	Nu13: 0.10
		E3: 10	G3: 25	Nu23: 0.10
Core	CF ply	E1: 18	G1: 3	Nu12: 0.25
		E2: 10	G2: 3	Nu13: 0.30
		E3: 3	G3: 2	Nu23: 0.30
	GF ply	E1: 6	G1: 2	Nu12: 0.25
		E2: 3	G2: 2	Nu13: 0.30
		E3: 2	G3: 2	Nu23: 0.30

**Table 2:** FEA model elements.

	Sandwich Composite	Loading Nose and Support Rollers
Element library	Standard	Standard
Geometric order	Linear	Linear
Family	Continuum Shell	3D Rigid
Element shape	Hex	Quad
Mesh size	1	1

**Table 3:** Benchmarking summary of structural supercapacitor performance.

Electrode	Separator	Electrolyte	Capacitance	Flexural Strength (MPa)	Temp Range (°C)	Ref.
CF	GF	DGEBA (Araldite GY 191)+ IL (EMITFSI) +LiTFSI- Double cell	57.26 mFcm^−2^	146	RT	This work
			114.5 mFcm^−2^	71	85	
CF	GF	DGEBA (Araldite GY 191)+ IL (EMITFSI) +LiTFSI- Double cell	1.16 mFcm^−2^	47.0	RT	[18]
			2.58 mFcm^−2^	14.2	85	
Activated CF	CP	Epoxy+1M TEABF4 in PC	25.4 mFg^−1^	29.1	RT	[49]
CF prepreg	GF	Epoxy+PVDF+ LiTf	11.62 mFg^−1^	47.5	RT	[50]
VG-MnO_2_-CF	GF	PEGDGE+IL (EMIMTFSI)+ LiTFSI	30.7 mFcm^−2^	32.0	RT	[51]

## Data Availability

The original contributions presented in this study are included in the article/Supplementary Material. Further inquiries can be directed to the corresponding author.

## Supplementary Materials

The following supporting information can be downloaded at: https://www.mdpi.com/article/10.3390/polym17172380/s1, Figure S1. Fabrication process (a) Hand lay-up using a roller, (b) Laminate placement in vacuum bag with applied vacuum pressure. Figure S2. GNP coated sandwich SSC with the configuration of CF-GNP/GF/GF/CF-GNP/GF/GF/CF-GNP/GF/GF/CF-GNP). Figure S3. Electrochemical testing (a) at room temperature (b) at elevated temperatures. Table S1. The specific capacitance at three different temperatures. Table S2. Hashin damage values defined for the FEA model. Figure S4. FEA model.
