# Supplementary material for: Functionalized Bisphenol A-Based Polymer for High-Performance Structural Supercapacitor Composites
Source: Polymers (Basel). 2025 Aug 31;17(17):2380. doi: 10.3390/polym17172380 (PMC12431032; doi:10.3390/polym17172380)
Supplement: Supplementary file 1 [file polymers-17-02380-s001.zip › polymers-3783832-supplementary.pdf]

Supporting Information

Preparation of GNP slurry and GNP coating on CF fabric

The GNP slurry containing 3 wt% GNP, 0.6 wt% PTFE and 0.2 wt% Triton-X in IPA solvent was prepared. PTFE was used as the binder and Triton X-100 was used as a surfactant. Using a tip sonicator (Sonoplus ultrasonic homogenisers, power 200 W) with 50% amplitude and 5 s on-off 5 s intervals, the slurry was sonicated for 30 minutes. Then, the GNP slurry was coated on both sides of CF fabric by using a spray gun at pressure around 3 bar, while maintaining a distance around 15 cm from the fabric. The GNP loadings were approximately 10 wt% based on the Eq. S1.

Calculation of GNP loading on CF fabric

$$\text{GNP loading} = \frac{(M_f - M_i)}{(M_i)} \times 100\% \quad \text{.................. Eq. S1}$$

Where M_i and M_f denote are the initial (bare CF) and final (after GNP slurry coated) mass of CFs respectively.

Fabrication of core

Structural supercapacitor core with double cell lay-up configuration were fabricated. Using hand lay-up method, the structural electrolyte was uniformly applied to CF and GF layers and fabricated the composite by curing at room temperature for 3 hours. Then, post-curing process was conducted in an oven at 85 °C for 3 hours.

Fabrication of Skin layers

(a) (b)

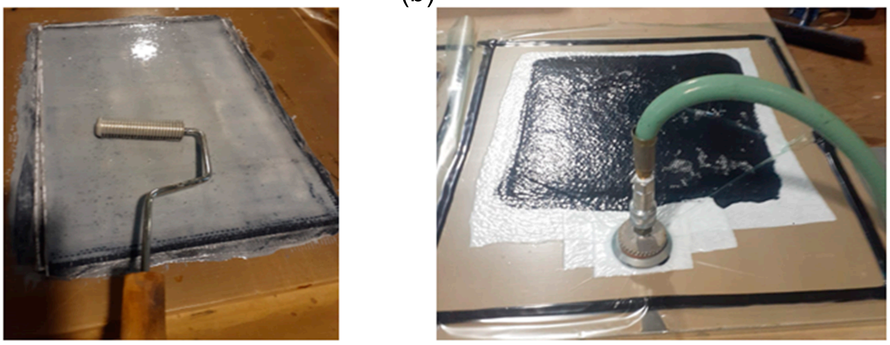

Figure S1: Fabrication process (a) Hand lay-up using a roller, (b) Laminate placement in vacuum bag with applied vacuum pressure

Fabrication of pouch type supercapacitor

The GNP-coated CF fabric was cut with the dimension of 50 mm × 80 mm, and a copper sheet was attached to its surface for electrochemical testing. Then, parallelly connected double cell SSC were fabricated as shown in Figure S1. The structural electrolyte mixture was evenly coated on CF electrodes and GF separators and mechanically pressed for curing at room temperature (25 ºC) for 48 hours. Then, it was heated for 3 hours in an oven at 85 °C for the post-curing process. The GNP uncoated part of the CF electrodes was not impregnated with the structural electrolyte to connect them to the potentiostat. The fabricated SSC was covered with two skin layers to manufacture sandwich SSC.

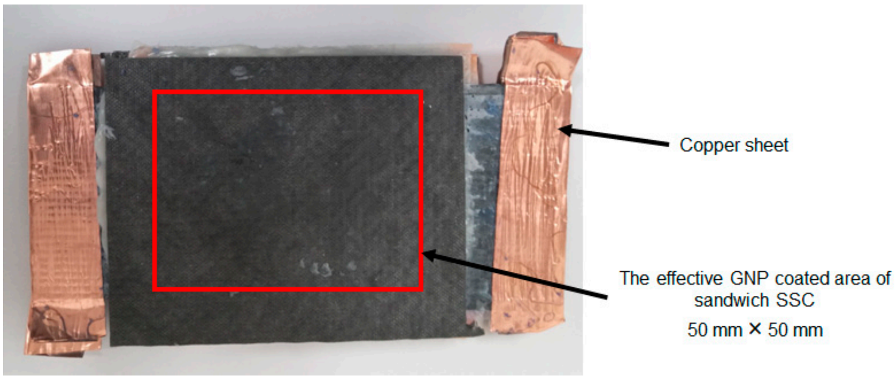

Figure S2: GNP coated sandwich SSC with the configuration of CF-GNP/GF/GF/CF-GNP/GF/GF/CF-GNP/GF/GF/CF-GNP)

Electrochemical performance

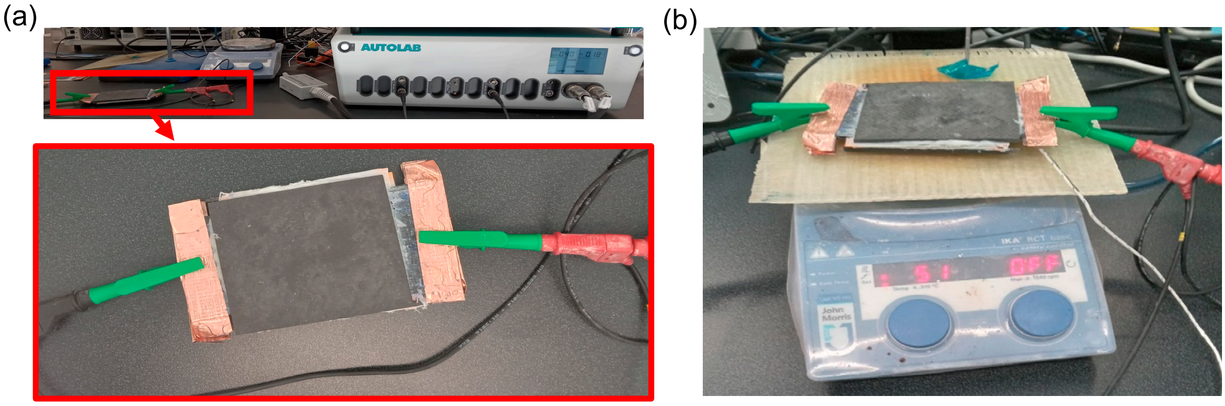

Figure S3: Electrochemical testing (a) at room temperature (b) at elevated temperatures

The equation for calculating specific capacitance calculation from the CV test:

$$C_{cv} = \int \frac{I\,dv}{v\,\Delta V} \quad \text{Eq. S2}$$

In the equation, C_{CV} (F) represents the capacitance, while ∫Idv denotes the area of the cyclic voltammogram corresponding to discharge process. The scan rate is υ (Vs^{-1}) and ΔV (V) is the potential window of the CV. In the case of calculating specific capacitance, the calculated capacitance was normalized to the weight of the supercapacitor, i.e., 50 g or normalized to the area of the supercapacitor, i.e., 25 cm^2.

The equation for calculating specific capacitance from the GCD test:

$$C_{GCD} = (I_d)\frac{t_d}{(\Delta V - V_{IR})} \quad \text{Eq S3}$$

In the equation, C_{GCD} (F) is the capacitance, while I_d (A) is the discharge current. t_d (s) is the discharge time, and ΔV (V) is the potential voltage of the GCD test. To ensure the accurate results, voltage drop due to internal resistance (IR) V_{IR} is excluded in GCD tests.

The equation for calculating energy density:

$$E = \frac{CV^2}{2\times3600} \quad \text{Eq S4}$$

In the equation, E (Wh) is the energy density, C (F) is the specific capacitance, and V is the potential window (V). For calculating areal specific energy density, the calculated capacitance was normalized to the weight of the supercapacitor or area of the supercapacitor.

Table S1: The specific capacitance at three different temperatures

**Temperature**	**$C_{sp,\,cv}$**	
	**At 5 $mV.s^{-1}$ scan rate**	**At 100 $mV.s^{-1}$ scan rate**
25 °C	801.36 mF/ 32.05 $mFcm^{-2}$	32.50 mF/ 1.30 $mFcm^{-2}$
65 °C	1480.89 mF/ 59.23 $mFcm^{-2}$	49.42 mF/ 1.97 $mFcm^{-2}$

85 °C	1846.93 mF/ 73.88 $mFcm^{-2}$	72.71 mF/ 2.90 $mFcm^{-2}$

Detail of FEA model

Table S2: Hashin damage values defined for the FEA model

Constituents		X_T (MPa)	X_C (MPa)	Y_T (MPa)	Y_C (MPa)	S_L (MPa)	S_T (MPa)
Skin		120	80	80	80	100	200
Core	CF ply	30	25	10	5	5	5
	GF ply	40	30	5	5	5	5

The notation X_T represents the longitudinal tensile strength, X_C for longitudinal compressive strength, Y_T for transverse tensile strength, Y_C for transverse compressive strength, S_L for longitudinal shear strength, and S_T for transverse shear strength.

Parts and assembly

The following model was used for the analysis. The skin layer was modelled as a composite with six CF lay-ups and core layer was modelled as 10 lay-ups [GNP coated CF/GF]$_s$. Two supporting noses were simply supported the specimen and lateral load was applied by the top loading cell at the center of laminates along y-axis. The supporting noses and loading nose were defined as the 3D discrete rigid. The details of FEA model elements are given in Table S2.

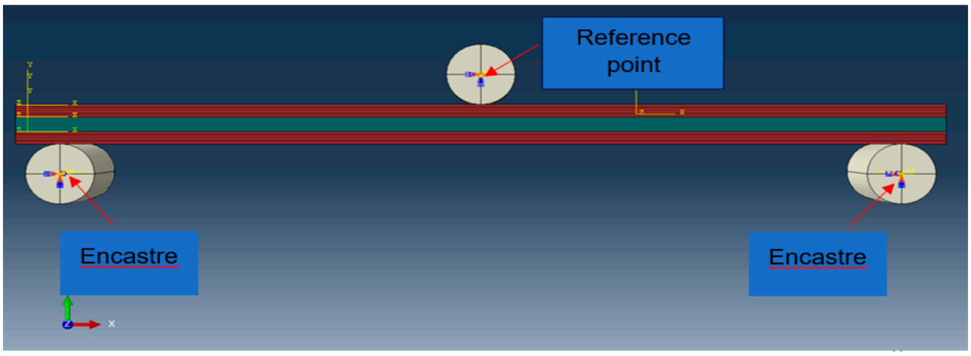

Figure S4: FEA model
